# PROMETHEUS: an observational, cross-sectional, retrospective study of hypertriglyceridemia in Russia

**DOI:** 10.1186/s12933-015-0268-2

**Published:** 2015-08-25

**Authors:** Yuri Karpov, Yunona Khomitskaya

**Affiliations:** Russian Cardiology Research and Production Complex, Moscow, Russian Federation; AstraZeneca, Moscow, Russian Federation

**Keywords:** Hypertriglyceridemia, Prevalence, Russia, Triglycerides, Glycated hemoglobin

## Abstract

**Background:**

Data regarding the prevalence of hypertriglyceridemia in the Russian population are lacking, despite triglyceride (TG)-mediated pathways being causal in cardiovascular disease. The prevalence of mixed dyslipidemia and severe hypertriglyceridemia in the Russian population (PROMETHEUS) was undertaken to address this gap.

**Methods:**

This was an observational, cross-sectional retrospective study. Data from adults with a full/partial lipoprotein record who had blood analyses done at an INVITRO laboratory in Russia between January 1, 2011 and December 31, 2013 were analyzed. The primary endpoint was the prevalence of hypertriglyceridemia (TG ≥ 1.7 mmol/L); secondary endpoints included prevalence of borderline high, high, and very high TG and severe hypertriglyceridemia, defined as a TG level of 1.7 to <2.3, 2.3 to <5.6, ≥5.6, and ≥10.0 mmol/L, respectively. Statistical analyses involved the Wilcoxon and the Chi square tests. Correlations between log-transformed TG and low- and high-density lipoprotein cholesterol (LDL-C and HDL-C) and total cholesterol (TC) were assessed. The correlation between glycated hemoglobin (HbA1c) and TG levels in a nested sample of subjects with HbA1c and TG data was also assessed using a log-linear model.

**Results:**

The full dataset and nested sample comprised 357,072 and 54,602 individuals, respectively. Prevalence of hypertriglyceridemia, borderline high TG, high TG, very high TG, and severe hypertriglyceridemia in the full dataset was 29.2, 16.2, 12.9, 0.11, and 0.011 %, respectively; corresponding rates in the nested sample were 19.0, 17.2, 0.25, and 0.016 %, respectively. TG levels were 16.4 % higher in males versus females; males had a greater risk of hypertriglyceridemia (risk ratio 1.25; 95 % CI 1.24, 1.26; P < 0.0001). Prevalence of hypertriglyceridemia increased with age, peaking at 40–49 years in males (42.8 %) and 60–69 years in females (34.4 %); a 0.61 % increase in TG levels for each year of life was predicted. Hypertriglyceridemia prevalence increased over time. Correlations between TG and LDL-C, HDL-C, TC, and HbA1c (nested sample only) were observed.

**Conclusions:**

Almost one-third of Russians have hypertriglyceridemia, but severe disease (TG ≥ 10.0 mmol/L) is rare. Although the risk of hypertriglyceridemia was greater in males versus females, its prevalence increased with age, regardless of sex. TG was associated with HbA1c, LDL-C, HDL-C, and TC.

## Background

Although there has been much debate over recent decades about the extent to which triglyceride (TG) levels directly promote cardiovascular disease [1], recent data indicate a causal role for TG-mediated pathways in cardiovascular disease, namely coronary artery disease [2, 3]. This is borne out in the results of several large-scale prospective cohort studies that have shown that elevated non-fasting TG levels are associated with an increased risk of cardiovascular events, including myocardial infarction, ischemic heart disease, and stroke [4–6]. As such, hypertriglyceridemia should be recognized as a prevalent and modifiable risk factor for cardiovascular disease that warrants treatment [3, 7].

A study examining the prevalence of hypertriglyceridemia in Russia is of particular interest due to the extremely high levels of cardiovascular and all-cause mortality in the country [8]. In 2012, the Russian life expectancy at birth was 75 years for women and 63 years for men [World Health Organization (WHO)], and between 2008 and 2013, the mortality rates per 1,000 were 367 for men and 137 for women [9]. These latter figures are approximately three times the mortality rates of men and women across the European region as a whole [9]. In 2014, more than 960,000 people died from circulatory system diseases in Russia [10].

A number of factors are known to influence TGs in the general population; these include (but are not limited to) obesity, physical inactivity, cigarette smoking, excess alcohol consumption, type 2 diabetes mellitus, certain drugs (e.g. estrogens), and specific genetic disorders [11]. Acute pancreatitis represents the most clinically relevant complication of hypertriglyceridemia [1], with up to 10 % of all cases being due to elevated TGs [12].

The results of various studies in countries such as Brazil, India (in middle-class urbanized individuals), Portugal, and the United States indicate that the overall prevalence of hypertriglyceridemia (i.e. a TG level of ≥1.7 mmol/L) is in the range of 21.6–33.5 % [13–17]. In Europe, the overall prevalence of hypertriglyceridemia has been reported as 29.6 % [18], while the prevalence of hypertriglyceridemia in individuals with coronary artery disease ranges between 21.1 and 44.6 % [19].

While the prevalence of hypertriglyceridemia has been well researched in some countries, there are a lack of data in the Russian population owing to a paucity of epidemiological studies and reliable registries. Data from a study in Northwest Russia indicate that 20.3 % of women and 22.6 % of men have a TG level ≥1.7 mmol/L [20]. In both women and men, the prevalence of hypertriglyceridemia was found to increase with age, starting at 6.1 and 12.0 %, respectively, in subjects aged 18–29 years, and peaking at 29.7 and 32.2 %, respectively, in subjects aged 50–59 years. Although this dataset reported the prevalence of other factors associated with metabolic syndrome, including glycated hemoglobin (HbA1c), it was not designed to analyze correlations between such factors and hypertriglyceridemia. In a separate study of 1561 urban office workers in St Petersburg (mean age 38.5 years), 12.6 % of women and 26.0 % of men had a TG level ≥1.7 mmol/L, with risk increasing with age. Hypertriglyceridemia was considered the fourth most prevalent component of metabolic syndrome following abdominal obesity, hypertension, and hyperglycemia, respectively, in this population [21]. In a study of older Muscovites aged 55 and older (mean age 67.6 years), 23.5 % of women and 22.1 % of men had a TG level ≥1.7 mmol/L; hypertriglyceridemia was deemed the rarest component of metabolic syndrome after hypertension, abdominal obesity, and decreased high-density lipoprotein cholesterol (HDL-C) for women, and hypertension and fasting hyperglycemia for men [22]. Although these datasets provide useful information about the prevalence of hypertriglyceridemia in Russia, they focus on either ethnically homogeneous populations in specific parts of the country or specific population cohorts, e.g. urban office workers, older individuals; furthermore, they do not include any information concerning the distribution of the different types of hypertriglyceridemia [20–22].

In the current report, the results of the Prevalence of mixed dyslipidemia and severe hypertriglyceridemia in the Russian population (PROMETHEUS) study are presented. The PROMETHEUS study was specifically undertaken to gather information on the prevalence of hypertriglyceridemia across the Russian Federation, including the prevalence of different severities and types of hypertriglyceridemia. The study also assessed the association between hypertriglyceridemia and other risk factors, namely HbA1c.

## Methods

### Study design

The PROMETHEUS study was an observational, non-interventional, cross-sectional retrospective study designed to determine the prevalence of hypertriglyceridemia in the Russian population. Because the study did not involve the collection of identifiable data and was retrospective, it was exempt from Ethics Committee review and informed consent was not obtained.

### Study population

Data for the PROMETHEUS study were extracted from an electronic database of subjects tested for blood lipids on an outpatient basis at Independent Laboratory INVITRO, the largest network of laboratories in the Russian Federation. INVITRO has medical offices in Russia, Ukraine, Belorussia, and Kazakhstan and is certified in accordance with ISO 15189:2007 and ISO 9001:2008. There are 638 INVITRO laboratory branches in 254 Russian cities, including Moscow and Saint Petersburg. In addition to the results of any laboratory tests, the INVITRO database holds information regarding the age and sex of all included individuals; however, no personal information that could disclose a participant’s identity is captured.

For the purposes of the current study, electronic data from male and female subjects aged ≥18 years with a full or partial lipoprotein record who had a blood analysis done at an INVITRO laboratory in Russia between January 1, 2011 and December 31, 2013 were extracted. A full or partial lipoprotein record included TG, total cholesterol (TC), HDL-C, and low-density lipoprotein cholesterol (LDL-C). Blood analysis conducted by INVITRO was undertaken in fasted patients using the following methodology: TC was determined using an enzymatic, colorimetric assay with cholesterol esterase and cholesterol oxidase (CHOD/PAP); HDL-C and TG levels were determined using homogeneous enzymatic colorimetric assays; and LDL-C was estimated indirectly from the measurements of TC, TG, and HDL-C using the Friedewald formula: [LDL-cholesterol] = [total cholesterol] − [HDL-cholesterol] − [triacylglycerol]/5 [23]. If the TG levels were higher than 4.5 mmol/L, LDL-C was measured using a direct enzymatic, colorimetric assay with cholesterol esterase and cholesterol oxidase. Estimation of inter- and intra-assay coefficients of variability was not possible because data were included from five INVITRO laboratory complexes over a period of 3 years. For each eligible subject, the last available lipoprotein analysis was extracted and written into the full dataset file; only one blood lipoprotein analysis was included for each individual and no follow-up was conducted. As part of the study, two datasets were analyzed: (1) the full dataset with lipoprotein data and (2) a nested sample from the full dataset with both lipoprotein and HbA1c data. To prevent duplication of both observations and individuals, new 32-point identification numbers were allocated to each individual when sampled. The study was wholly observational and there were no interventions relating to routine clinical practice, either in terms of therapy or special examinations.

### Study outcomes

The primary objective of the PROMETHEUS study was to estimate the percentage of subjects with hypertriglyceridemia (serum TG level ≥1.7 mmol/L). Secondary objectives were as follows: (1) estimated proportion of subjects with borderline high serum TG (1.7 to <2.3 mmol/L), high serum TG (2.3 to <5.6 mmol/L), and very high serum TG (≥5.6 mmol/L), as per the Adult Treatment Panel (ATP) III guidelines of the National Cholesterol Education Program (NCEP) [11]; (2) estimated proportion of subjects with severe hypertriglyceridemia (serum TG ≥ 10.0 mmol/L), as per the European Atherosclerosis Society (EAS) consensus panel [24]; and (3) estimated proportion of subjects with mixed hyperlipoproteinemia (i.e. Fredrickson type 2b classification comprising elevated levels of TG, TC, and LDL-C [24] [≥1.7, ≥5.2, and ≥3.4 mmol/L, respectively, for the purposes of the study]). Other secondary endpoints, assessed only in the nested sample, were the estimated correlations between (1) HbA1c and TG levels and (2) a high HbA1c level (≥6.5 %) and a high TG level (≥1.7 mmol/L). (An HbA1c of 6.5 % is recommended as the cut point for diagnosing diabetes, as per World Health Organization criteria [25].) Data for the primary and secondary endpoints were retrieved wholly from the INVITRO database. No information for potential confounders or effect modifiers was available.

### Statistical methods

The number of eligible subjects within the entire INVITRO database determined the size of the sample. Standard descriptive parameters were calculated for all continuous variables. The estimated proportions (and associated 95 % confidence intervals [CIs]) of subjects with hypertriglyceridemia, borderline high TG, high TG, very high TG, and severe hypertriglyceridemia were calculated in both the full dataset and in the nested dataset. The Wilcoxon test and the Chi square test were used to compare differences in mean age and the proportion of males to females, respectively, in the full dataset and in the nested sample. For the most part, statistical test data are not included in the current report due to the extremely large dataset (i.e. owing to the large number of study participants, even slight differences might appear to be statistically significant, even when they have no clinical relevance).

Stratified analyses that examined the prevalence of hypertriglyceridemia according to sex (male and female), age (18–29, 30–39, 40–49, 50–59, 60–69, 70–79, 80–89, and ≥90 years) and study year (2011, 2012, and 2013) were performed; linear regression was undertaken to assess annual changes in the prevalence of hypertriglyceridemia from 2011 to 2013. Within the full dataset, models were used to assess the correlation between TG and (1) TC (log[TG] = β_0_ + β_1_*log[TC]), (2) LDL-C (log[TG] = β_0_ + β_1_*log[LDL-C]), and (3) HDL-C (log[TG] = β_0_ + β_1_*log[HDL-C]). The correlation between HbA1c and TG levels in the nested sample was assessed in the following log-linear model: log(TG) = β_0_ + β_1_*HbA1c. In all instances, the models were used to predict TG level change as a result of a one-unit change in the independent variable while all the predictors were held constant.

Statistical analyses were performed using those observations with no missing values in any of the mentioned variables. In the case of missing data, separate analyses were conducted and compared with the non-missing data population to establish whether the values were missed at random or had some pattern (i.e. to establish whether missing data bias could be introduced into the estimates).

## Results

**Fig. 1:**
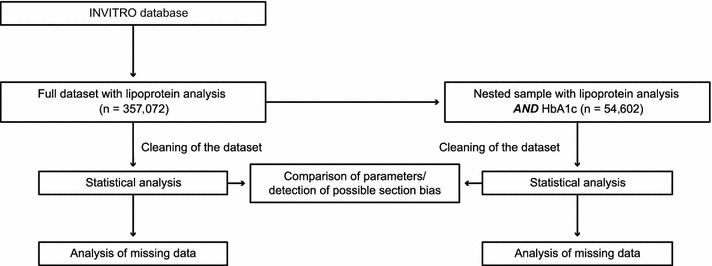
Extraction and analysis of data from the INVITRO database. *HbA1c* glycated hemoglobin.

A total of 357,073 individuals were identified as being eligible for inclusion in the PROMETHEUS study; one of the observations, however, was dated 2010 and was excluded. As such, 357,072 individuals formed the full dataset, while 54,602 individuals in the full dataset had both lipoprotein and HbA1c data and formed the nested sample (Fig. 1). Fifty-one observations lacked a sex identifier; sex values for these individuals were marked as missing.

### Demographic information

The overall proportion of males was 38.4 % (n = 137,139) in the full dataset and 38.3 % (n = 20,908) in the nested sample. The mean age of male and female participants was 48.2 and 51.0 years, respectively, in the full dataset and 49.8 and 51.9 years, respectively, in the nested sample.

### Prevalence of hypertriglyceridemia

**Fig. 2:**
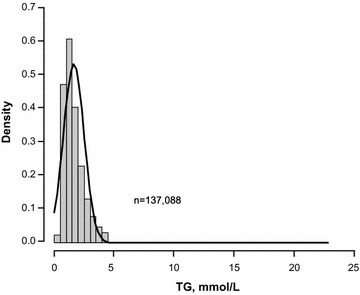
Histogram for TG distribution, males. *TG* triglycerides.

**Fig. 3:**
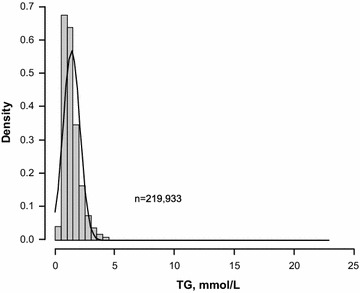
Histogram for TG distribution, females. *TG* triglycerides.

Overall, 29.2 % (n = 104,299; 95 % CI 29.1, 29.4 %) of subjects in the full dataset had hypertriglyceridemia, defined as a serum TG level ≥1.7 mmol/L. Of the 104,299 subjects with hypertriglyceridemia in the full dataset, 55.4 % (n = 57,794) had borderline high TG, 44.2 % (n = 46,102) had high TG, 0.39 % (n = 403) had very high TG (all as per the NCEP ATP III guidelines), and 0.037 % (n = 39) had severe hypertriglyceridemia (as per the EAS consensus panel); the corresponding proportions in the 19,906 subjects in the nested sample with hypertriglyceridemia were 52.1 % (n = 10,377), 47.2 % (n = 9,395), 0.67 % (n = 134), and 0.045 % (n = 9), respectively. The distributions of TGs in men and women in the full dataset are shown in Figs. 2 and 3; the prevalence of hypertriglyceridemia across both datasets is shown in Table 1.

**Table 1:** Prevalence of hypertriglyceridemia (full dataset and nested sample)

Parameter, n, % (95 % CI)	Full dataset (n = 357,072)	Nested sample (n = 54,602)
Hypertriglyceridemia (TG ≥ 1.7 mmol/L)	104,299	19,906
	29.2 (29.1, 29.4)	36.5 (36.1, 36.9)
Borderline high serum TG (≥1.7 to <2.3 mmol/L)	57,794	10,377
	16.2 (16.1, 16.3)	19.0 (18.7, 19.3)
High serum TG (≥2.3 to <5.6 mmol/L)	46,102	9,395
	12.9 (12.8, 13.0)	17.2 (16.9, 17.5)
Very high serum TG (≥5.6 mmol/L)	403	134
	0.11 (0.10, 0.12)	0.25 (0.21, 0.29)
Severe hypertriglyceridemia (TG ≥ 10.0 mmol/L)	39	9
	0.011 (0.008, 0.015)	0.016 (0.008, 0.031)

*CI* confidence interval, *TG* triglycerides.

**Fig. 4:**
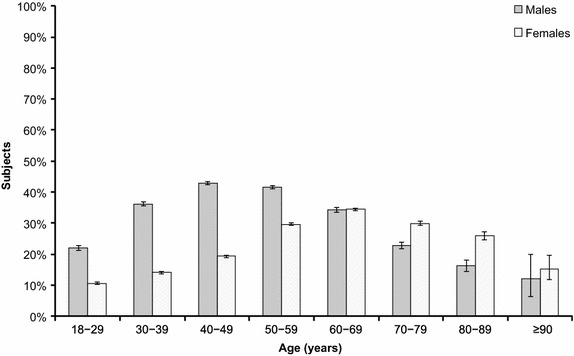
Prevalence of hypertriglyceridemia according to age and sex (full dataset). Full dataset (n = 357,072). Data shown as percentage (95 % confidence interval).

TG levels were 16.4 % higher in males than in females and the risk of hypertriglyceridemia was also higher in males (risk ratio [RR] 1.25; 95 % CI 1.24, 1.26; P < 0.0001). In males, the prevalence of hypertriglyceridemia increased from 22.0 % at 18–29 years to 42.8 % at 40–49 years, decreasing to 12.0 % at age ≥90 years (Figs. 2, 3). In females, the prevalence of hypertriglyceridemia increased from 10.6 % at age 18–29 years, peaking at 34.4 % at age 60–69 years; thereafter, the prevalence decreased to 15.4 % in subjects aged ≥90 years (Fig. 4). A 0.61 % increase in TG levels for each year of life was predicted (P < 0.0001).

**Fig. 5:**
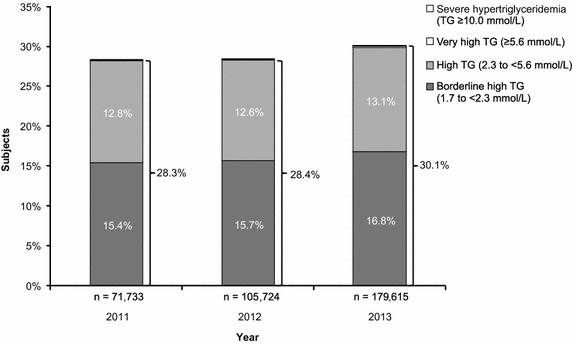
Prevalence of borderline high TG, high TG, very high TG, and severe hypertriglyceridemia by year. Full dataset (n = 357,072). The overall prevalence of hypertriglyceridemia (TG level ≥1.7 mmol/L) is shown to the *right* of each *bar*. The prevalence of very high TG in 2011, 2012, and 2013 was 0.03 % (n = 25), 0.08 % (n = 84), and 0.16 % (n = 294), respectively. The prevalence of severe hypertriglyceridemia in 2011, 2012, and 2013 was 0.0042 % (n = 3), 0.0066 % (n = 7), and 0.016 % (n = 29), respectively. Increases were significant (P < 0.0001 for hypertriglyceridemia, *borderline* high TG, and very high TG; P < 0.01 for high TG; P < 0.05 for severe hypertriglyceridemia). *TG* triglycerides.

Overall, 71,733, 105,724, and 179,615 subjects were analyzed in 2011, 2012, and 2013, respectively. There was an increase in the prevalence of hypertriglyceridemia from 2011 to 2013 (28.3 % [n = 20,279], 28.4 % [n = 30,011], and 30.1 % [n = 54,009] in 2011, 2012, and 2013, respectively [P < 0.0001]) (Fig. 5). The observed increase in prevalence from 2011 to 2013 was apparent for all degrees of severity of hypertriglyceridemia (Fig. 5); notably, the prevalence of severe hypertriglyceridemia was 0.0042 % (n = 3), 0.0066 % (n = 7), and 0.016 % (n = 29) in 2011, 2012, and 2013, respectively (P < 0.05).

### Prevalence of hypertriglyceridemia with concomitant mixed hyperlipoproteinemia

In the full dataset, the proportion of subjects with mixed hyperlipoproteinemia (TG ≥ 1.7 mmol/L, TC ≥ 5.2 mmol/L and LDL-C ≥ 3.4 mmol/L) was 19.2 % (95 % CI 19.1, 19.3 %); the corresponding proportion in the nested sample was 21.8 % (95 % CI 21.4, 22.1 %).

### Correlation between triglyceride levels and glycated hemoglobin

**Fig. 6:**
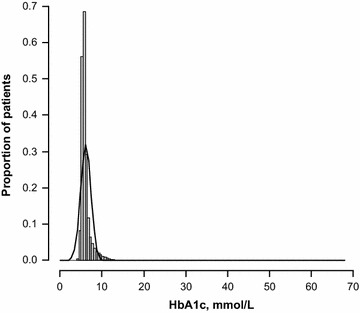
Glycated hemoglobin (HbA1c) distribution prior to log transformation. HbA1c distribution did not improve its shape after log transformation.

**Fig. 7:**
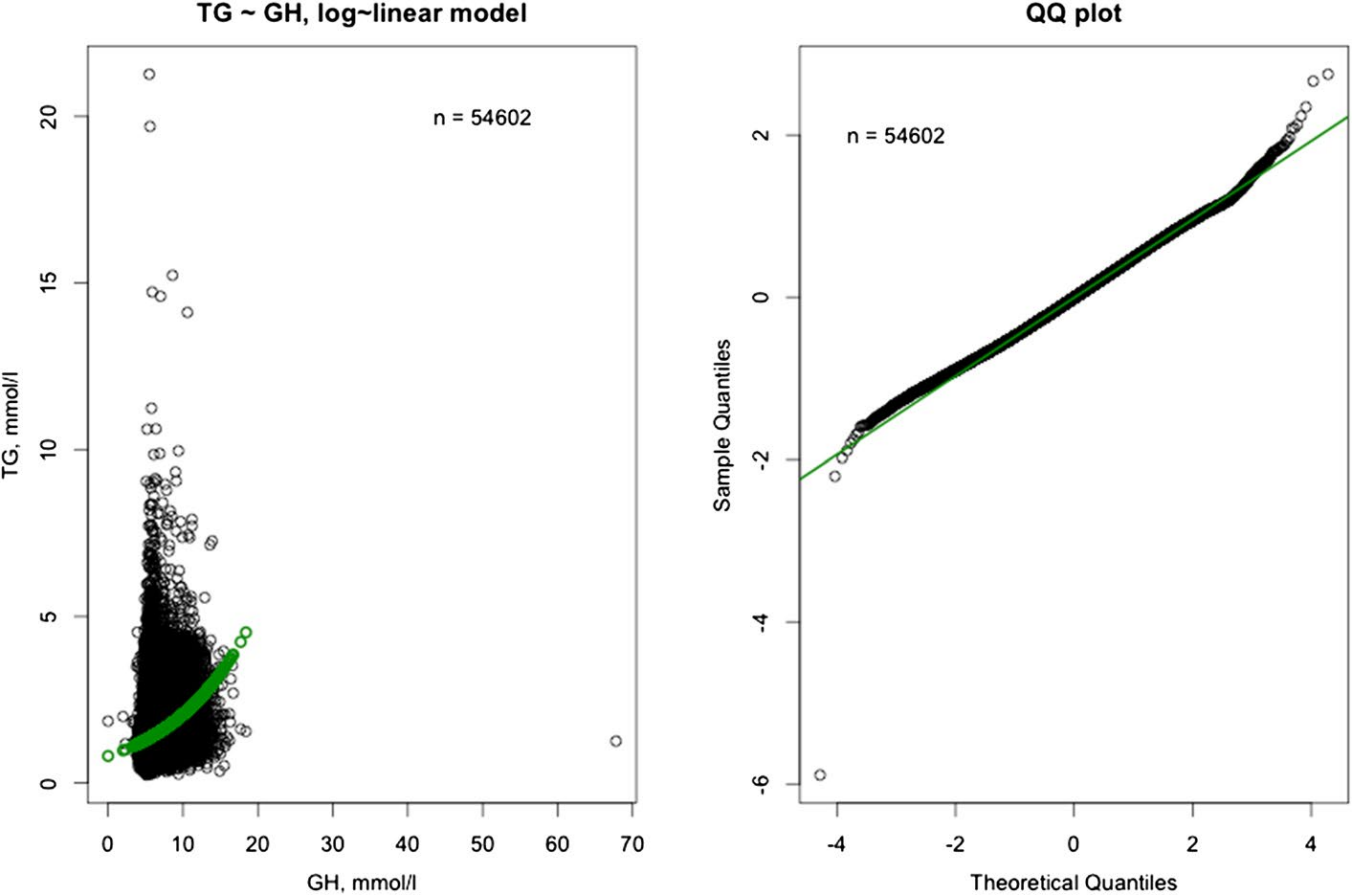
Visualization of the model: log(TG) = β_0_ + β_1_*GH. Nested sample (n = 54,602). *TG* triglycerides, *GH* glycated hemoglobin. The model is a good fit with the real data.

In the nested sample (i.e. subjects in the full dataset with lipoprotein and HbA1c data [n = 54,602]), the prevalence of a high HbA1c level (≥6.5 %) was 11.0 % (95 % CI 10.7, 11.2 %); the HbA1c distribution prior to log transformation is shown in Fig. 6. Correlation between HbA1c and TG was assessed using the log-linear model: log(TG) = β0 + β1*GH. The positive correlation between HbA1c and TG (R = 0.0933) predicted a 9.3 % increase in TG level with each one-unit increase in HbA1c level (P < 0.0001). The risk of a high TG level was greater in the presence versus the absence of high HbA1c (RR 1.69; 95 % CI 1.65, 1.72). Conversely, the risk of a high HbA1c level was greater in the presence versus the absence of a high TG level (RR 2.04; 95 % CI 1.98, 2.11) (Table 2). The relationship between TG levels and HbA1c levels is shown in Fig. 7.

**Table 2:** Relationship between high glycated hemoglobin (HbA1c) levels and high triglyceride (TG) levels (nested sample)

n	TG ≥ 1.7 mmol/L	TG < 1.7 mmol/L	Total	Risk ratio (95 % confidence interval)	
				HbA1c/TG	TG/HbA1c
HbA1c ≥6.5 %	5,995	5,112	11,107	1.69 (1.65, 1.72)	2.04 (1.98, 2.11)
HbA1c <6.5 %	13,911	29,581	43,492		
Total	19,906	34,693	54,599

### Correlation between triglyceride levels and other lipids

**Fig. 8:**
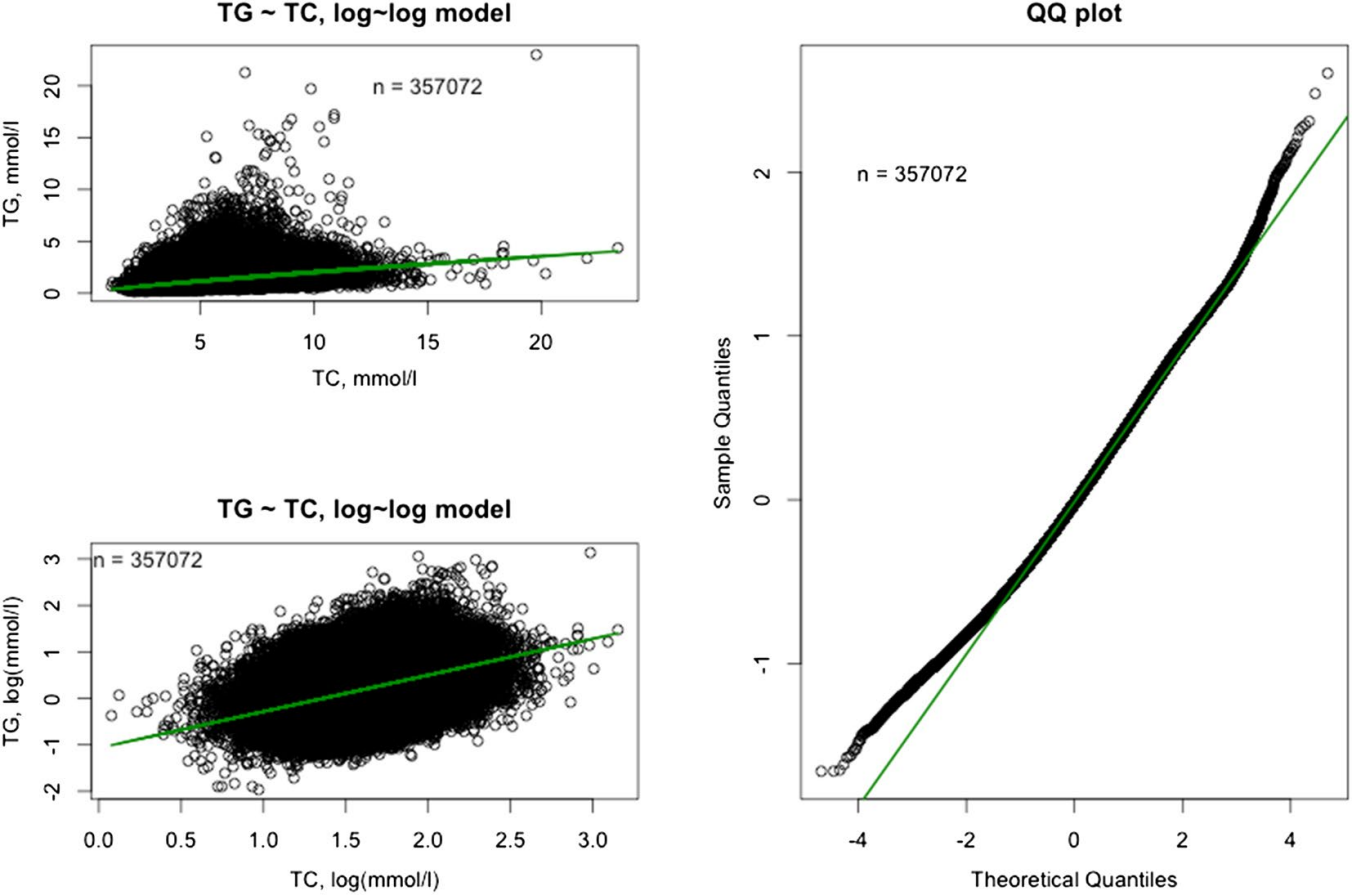
Visualization of the model: log(TG) = β_0_ + β_1_*log(TC). Full dataset (n = 357,072). The model is a good fit with the real data. *TG* triglycerides, *TC* total cholesterol.

**Fig. 9:**
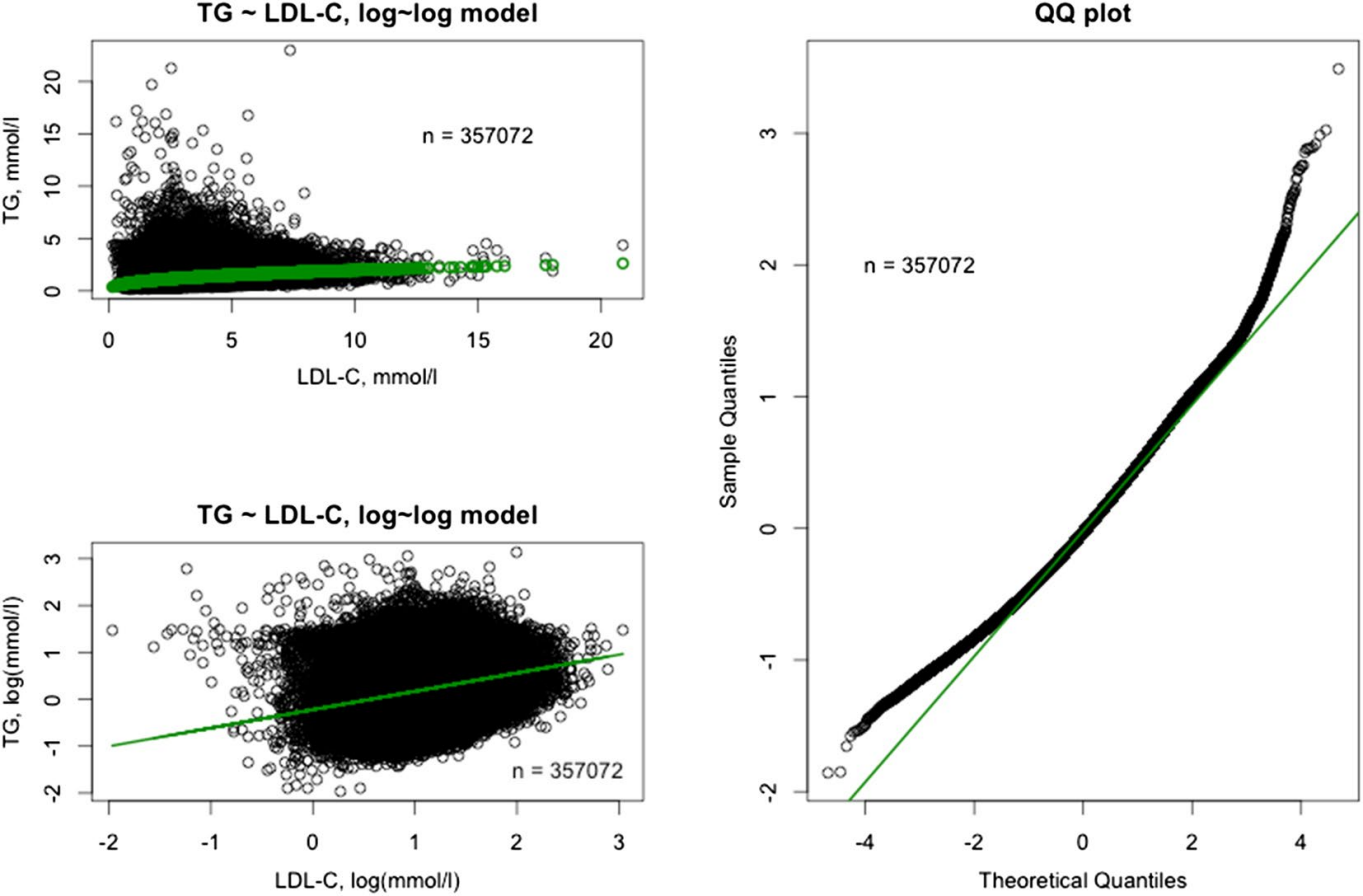
Visualization of the model: log(TG) = β_0_ + β_1_*log(LDL-C). Full dataset (n = 357,072). The model is a good fit with the real data. *TG* triglycerides, *LDL-C* low-density lipoprotein cholesterol.

**Fig. 10:**
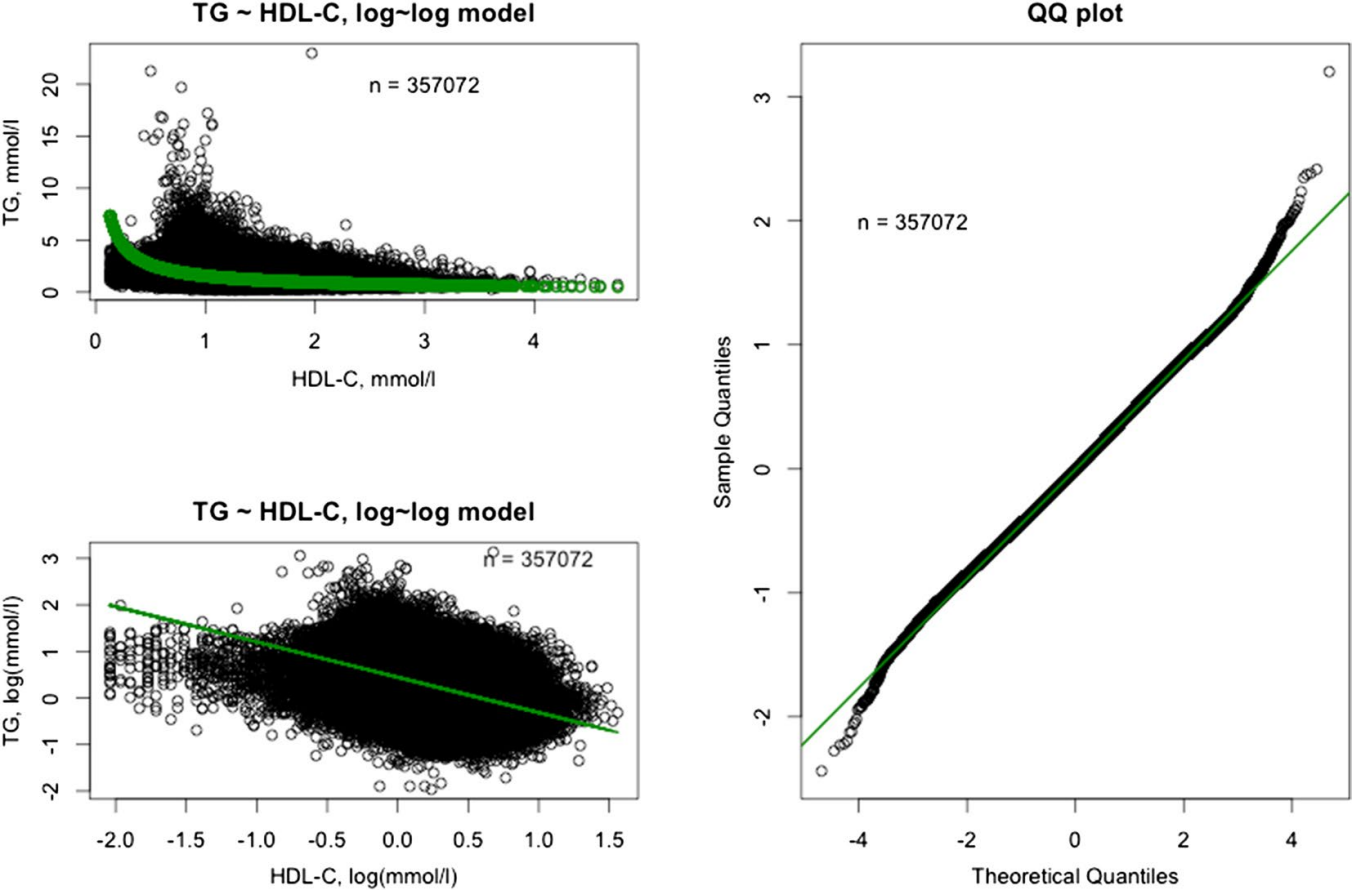
Visualization of the model: log(TG) = β_0_ + β_1_*log(HDL-C). Full dataset (n = 357,072). The model is a good fit with the real data. *TG* triglycerides, *HDL-C* high-density lipoprotein cholesterol.

The relationships between TG levels and other lipids are depicted in Figs. 8, 9, 10. In the full dataset and in the nested sample, mean LDL-C levels were 3.63 and 3.54 mmol/L, respectively, mean HDL-C levels were 1.32 and 1.25 mmol/L, respectively, and mean TC levels were 5.61 and 5.52 mmol/L, respectively. The correlation between LDL-C and TG was assessed using the log-linear model: log(TG) = β_0_ + β_1_*log(LDL-C). Low correlations (full dataset, R = 0.3909; nested sample, R = 0.2658) were found with the model predicting a 0.39 % and a 0.27 % increase in TG level with each 1 % increase in LDL-C level in the full dataset and nested sample, respectively (P < 0.0001). The correlation between TC and TG was assessed using the log-linear model: log(TG) = β_0_ + β_1_*log(TC). The model predicts a 0.78 % increase in TG level with each 1 % increase in TC level (P < 0.0001). The determination coefficient for the model is 0.13 and accounts for 13 % of all variability in TG levels. The correlation between HDL-C and TG was assessed using the log-linear model: log(TG) = β_0_ + β_1_*log(HDL-C). The model predicts a 0.76 % decrease in TG level with each 1 % increase in HDL-C level (P < 0.0001). The determination coefficient for the model is 0.18 and accounts for 18 % of all variability in TG levels (Table 3).

**Table 3:** Linear models of log transformed triglyceride (TG) levels

Log(TG) ~	Full dataset, n = 357,072			Nested sample, n = 54,602		
	Coefficient	P value	R^2^	Coefficient	P value	R^2^
Model 1						
Age	0.0061	<0.0001	0.03	0.0049	<0.0001	0.02
Model 2						
Sex (f)	Reference	–	–	Reference	–	–
Sex (m)	0.1637	<0.0001	0.03	0.1574	<0.0001	0.03
Model 3						
Log(TC)	0.7837	<0.0001	0.13	0.7193	<0.0001	0.11
Model 4						
Log(LDL-C)	0.3909	<0.0001	0.06	0.2658	<0.0001	0.03
Model 5						
Log(HDL-C)	−0.7588	<0.0001	0.18	−0.8110	<0.0001	0.19
Model 6						
HbA1c	–	–	–	0.0933	<0.0001	0.06

### Analysis of missing data

A comparison of observations with missing values to the non-missing-data population did not identify any pattern that might introduce missing data bias into the estimates.

## Discussion

The large-scale PROMETHEUS study was designed to estimate the proportion of subjects with hypertriglyceridemia in the Russian Federation using data captured within a nationwide database over a 3-year period. The results of the study show that almost one-third of the Russian population has hypertriglyceridemia, defined as a TG level ≥1.7 mmol/L. Men were found to have a higher risk for hypertriglyceridemia than women, although, with both sexes, the prevalence of hypertriglyceridemia increased with age. TG levels were found to be associated with levels of TC, LDL-C, and HDL-C. An analysis of data in a nested sample of subjects with TG and HbA1c data demonstrated a linear association between a high HbA1c level and a high TG level.

The overall rate of hypertriglyceridemia in the current study (29.2 %) is within the range reported in several other studies [13–18]. In a collaborative analysis of nine European population-based cohorts that included 10,269 adults (mean age, 46–62 years; % male, 41.4–51.0 %), 29.6 % of participants had elevated TGs (≥1.7 mmol/L) [18]. Data from country-specific studies conducted within Europe (e.g. Italy, Norway, Portugal, Spain) showed that the prevalence of hypertriglyceridemia (defined as a TG level ≥1.7 mmol/L) ranged from 13 to 33.3 %, depending on gender [14, 26–28]. Data taken from six of the 2-year National Health and Nutrition Examination Survey (NHANES) cycles (from 1999–2000 to 2009–2010) showed that between 24.3 and 33.5 % of the adult population (aged ≥ 20 years) in the United States had a TG level ≥1.7 mmol/L [13]. Interestingly, the rate of hypertriglyceridemia in the current study was somewhat higher than in other studies conducted in Russian populations. In three studies of urban populations in Russia, the prevalence of hypertriglyceridemia (TG level ≥1.7 mmol/L) ranged from 12.6 to 23.5 % for women and 22.1 to 26.0 % for men, depending on age [20–22]. However, the demographically diverse populations included in the nationwide dataset from INVITRO may account for the higher prevalence of hypertriglyceridemia recorded in this study. For example, in a small study that investigated fatty acid composition and food consumption among Izhma reindeer herders (practicing a traditional lifestyle) and urban inhabitants of the city of Syktyvkar in Northern Russia, significantly higher levels of TC, TG, and HDL-C were observed in male reindeer herders in comparison with male urban citizens [29]. Thus the disparate lifestyles and nutritional habits of different populations across Russia may result in a higher overall prevalence of hypertriglyceridemia. Gender differences were noted in our study. First, the prevalence of hypertriglyceridemia was greater in men than in women, with men being 25 % more likely to have hypertriglyceridemia than women. The higher rate of hypertriglyceridemia in men than women in the PROMETHEUS study is in agreement with other studies that have assessed between-sex differences in the rates of hypertriglyceridemia [18, 26–28]. Additional data from the NHANES (1999–2002) indicate that serum TGs are significantly higher in men than in women in the United States [30]. This is despite the fact that the use of oral contraceptives and hormone replacement therapy can increase TG concentrations in women [15].

Second, in both men and women, the prevalence of hypertriglyceridemia rose with increasing age, but peaked in men aged 40–49 years and in women aged 60–69 years. The later peak in women may be explained by the known increase in TG levels that occurs during the menopause [31]. The finding that the prevalence of hypertriglyceridemia increases with age and is higher in men than in women mirrors what has been reported for cardiovascular disease (e.g. coronary heart disease and myocardial infarction) in both the United States and Europe [32, 33].

It is plausible that both alcohol consumption and obesity have contributed to the high prevalence of hypertriglyceridemia among Russian men in this study. Alcohol consumption among Russian men (23.9 L of pure alcohol per person per annum in 2010) is more than double the average of European member states (10.9 L per person per annum), while Russian women consume considerably less at just 7.8 L of pure alcohol per annum [8, 34]. From 2010 to 2014 the prevalence of obesity (aged ≥18 years; body mass index [BMI] ≥30) in Russia rose from 17.6 to 20.3 % for men and from 26.2 to 27.4 % for women, in line with the trend across other European countries [35]. While the impact of alcohol consumption on TG levels is not fully understood, recent studies have shown that excessive (over 210 g/week for men; over 140 g/week for women), but not moderate alcohol consumption (up to 210 g/week for men; up to 140 g/week for women), may contribute to a rise in TG levels [36–38]. In a study of 1,477 men with diabetes, the log-transformed TG/HDL-C ratio was significantly lower in light and moderate drinkers versus non-drinkers, but there was no significant difference between heavy drinkers and non-drinkers [39]. Bessembinders et al. noted that nearly a quarter of patients with severe hypertriglyceridemia (TG levels ≥11.3 mmol/L) met the criteria for excessive consumption of alcohol; moreover, 43 % of those who excessively consumed alcohol were in the highest TG quartile [36]. Obesity, diabetes, and a diet high in saturated fat further exacerbate the effect of alcohol consumption on TG levels, leading to hypertriglyceridemia not only postprandially, but also in the fasting state [36, 38].

Over the course of the study, an increase in the prevalence of hypertriglyceridemia was observed. Given the short time frame of the study (3 years), this finding might simply be due to chance. Conversely, it may be explained by changes in lifestyle factors on the part of the participants, including diet, smoking, and physical activity, although it is not possible to account for such factors within the current study. Differing results regarding change over time in TG levels have been reported in previous studies. One study reported that TG levels have increased over the past 20 years in both men and women in the United States, with increases being most noticeable in younger age groups (i.e. individuals aged 20–49 years) [1]. Conversely, data collected over a 12-year period as part of the NHANES showed that the prevalence of hypertriglyceridemia significantly decreased from 33.5 % in 1999–2000 to 24.3 % in 2009–2010 in the United States [13]; this was accompanied by a significant decrease in the mean serum TG level, from 123 mg/dL (1.39 mmol/L) in 1999–2002 to 110 mg/dL (1.24 mmol/L) in 2007–2010 [40]. Another analysis of NHANES data, comparing the period 2005–2008 with 1988–1991, also reported significant reductions in TG levels, though only among individuals with undiagnosed diabetes or prediabetes after adjustment for demographic characteristics [41]. Among the populations with diagnosed diabetes and normoglycemia, trends towards reductions in TG levels did not reach statistical significance. Decreases in the prevalence of hypertriglyceridemia and in mean TG levels in the NHANES dataset are likely to have resulted from increased uptake of lipid-modifying drugs, including statins [13, 40, 41]. Owing to the type of information included in the INVITRO database, no insight into the uptake and effect of lipid-modifying medications in the current study was possible.

The PROMETHEUS study included a separate analysis of outcomes in a sample of patients with both lipoprotein and HbA1c data. Within this nested sample, the correlation between HbA1c and TG predicted a 9.3 % increase in TG level with each one-unit increase in HbA1c level. This correlation is not unexpected given that the HbA1c test is conducted in individuals who have, or are suspected to have, type 2 diabetes mellitus (T2DM), and hypertriglyceridemia is frequently seen in patients with diabetes and in those with metabolic syndrome [42]. Indeed, the known relationship between TGs and fasting glucose has prompted the recommendation that hypertriglyceridemic states be promptly followed up to rule out the presence of T2DM [1]. Data from a study conducted in a middle-class urban population in India showed that the age-adjusted prevalence of hypertriglyceridemia was 71.0 versus 30.2 % in the presence versus the absence of diabetes [17]. Additionally, data from a cross-sectional study conducted in Turkey showed that mean serum levels of TG were significantly higher in patients with diabetes compared with those without [43].

The results of the PROMETHEUS study showed a correlation between TG and other lipid parameters. Namely, increases in the levels of TC and LDL-C and decreases in the level of HDL-C were predicted to result in significant increases in TG. These results demonstrate the complex relationship between the levels of TG and lipoproteins, all of which are modifiable risk factors for cardiovascular disease [44]. The Residual Risk Reduction Initiative (R^3^I) has identified atherogenic dyslipidemia (imbalance between proatherogenic triglyceride-rich apolipoprotein B-containing lipoproteins and antiatherogenic apolipoprotein A-I-lipoproteins) as an important modifiable contributor to residual cardiovascular risk, and highlighted the need for education to improve awareness of this condition [45]. Assessment of non-HDL cholesterol has been proposed by the R^3^I as a basis for determining treatment of lipid-related residual cardiovascular risk [45].

Current treatment protocols for patients with dyslipoproteinemias, including those with T2DM, focus primarily on lowering LDL-C or raising HDL-C in order to reduce a patient’s risk of cardiovascular disease. While statins have been shown to be effective in lowering LDL-C and levels of triglyceride-rich lipoproteins [46, 47], the residual risk of vascular events remains high in this patient group. The extent to which this residual risk is attributable to persistent lipoprotein abnormalities remains to be determined; however, two recent studies have emphasized elevated TG levels as a significant risk factor. A longitudinal, observational study of 1917 T2DM patients, with a mean follow-up period of 10 years, demonstrated a direct association between mean TG levels and long-term mortality risk in older T2DM patients, even after taking into account traditional cardiovascular risk factors (BMI, HbA1c, and LDL-C) and pharmacological treatments [48]. In a separate study of patients with acute coronary syndrome who were receiving treatment with statins, fasting TG levels were found to strongly predict both long-term and short-term risk of recurrent ischemic events in this population even after adjustment for risk factors (age, sex, hypertension, smoking, diabetes, HDL-C, BMI, and LDL-C) [49]. These findings suggest a potential role for the therapeutic management of TG-rich lipoproteins. Fibrates such as bezafibrate have been shown to lower TG levels in patients with metabolic syndrome and offer potential vascular protective effects in these patients [3, 45, 50]. Ezetimibe may also produce small reductions in TG levels, and both fibrates and ezetimibe can be administered in combination with statins for broader treatment of dyslipidemia [3, 45]. In addition, the TG lowering effects of prescription omega 3 fatty acids (PO3FA) are well established with recent clinical trials, demonstrating that the administration of a daily dose of 4 g PO3FA in patients with very high TG levels (≥500 mg/dL) can reduce TG by 27–45 % [51–53]. While the impact of PO3FA on cardiovascular outcomes has yet to be fully established, a large-scale randomised placebo-controlled PO3FA intervention trial [54] and a long-term outcomes trial [55] are currently underway to evaluate whether appropriate prescription-strength dosing of PO3FA can reduce cardiovascular events when used as an adjunct to statin therapy in patients with persistently high TG and at high risk of cardiac events. Nevertheless, there remains a need for improved treatment and novel options such as next-generation peroxisome proliferator-activated receptor (PPAR) agonists, cholesteryl ester transfer protein (CETP) inhibitors, proprotein convertase subtilisin/kexin type 9 (PCSK9)-targeted therapy, and apolipoprotein A-I therapies are being explored [45].

Within the PROMETHEUS study, fewer than 1 % of participants had either very high TG or severe hypertriglyceridemia. Studies using data from cycles of the NHANES reported a rate of prevalence of 1.7 % for TG ≥ 500 mg/dL (≥5.6 mmol/L), 0.4 % for TG ≥ 1,000 mg/dL (≥11.2 mmol/L) [15], and 1.7 % for TG of 500–2,000 mg/dL (5.6–22.6 mmol/L) [56]. The low rate of severe disease in the current study is somewhat encouraging given the known relationship between severe hypertriglyceridemia and acute pancreatitis. Nevertheless, despite the Endocrine Society Clinical Practice Guidelines (2012) noting that only mild or moderate hypertriglyceridemia are considered risk factors for cardiovascular disease [57], epidemiological data are increasingly pointing towards severe hypertriglyceridemia as a risk factor for increased long-term total mortality and cardiovascular risk; thus patients with all severities of the condition may be affected and ought to be treated accordingly [48, 58].

There are a number of limitations of the current analysis that deserve mention. First, the INVITRO laboratory generally works with outpatients and such individuals are more likely than the general population to have cardiovascular disorders, diabetes, or other acute and chronic disorders associated with metabolic syndrome or transiently altered lipoprotein levels; this would result in selection bias moving prevalence ratio estimates away from the null. Second, there was a lack of key variables in the dataset (e.g. main presenting condition, region of the country, bodyweight, socioeconomic status, diabetes status, alcohol consumption, use of lipid-lowering medications, etc.) that could be used to control for confounding factors in the calculation of the prevalence of hypertriglyceridemia. Third, while estimation of inter- and intra-assay coefficients of variability is standard practice for INVITRO laboratories, it wasn’t possible in this instance because the PROMETHEUS study dataset utilized data from five INVITRO laboratory complexes for a period of 3 years.

Fourth, as is general practice, TG levels were established in fasted patients; however, evidence from recent epidemiologic studies suggests that non-fasting TGs may be a better predictor of cardiovascular disease [4, 6]. Fifth, because there was no way to ascertain the region of the country that participants were from, it was not possible to capture any patterns in dyslipoproteinemias that might exist in what is a vast geographical area. Last, the lack of descriptive variables in the dataset leaves unanswered questions about the importance of confounders and effect modifiers (e.g. diabetic status, alcohol consumption, pregnancy, etc.); this might decrease generalizability of the outcomes. Such limitations, however, should not detract from the strengths of the study, namely the large number of participants and the utilization of up-to-date data from laboratories with a wide geographical reach. This work was performed to draw attention to the problem of hypertriglyceridemia, which is often not within the remit of the healthcare physician. It appears that, to date, the prevalence of hypertriglyceridemia has been underestimated in Russia; thus, this study provides a platform for future confirmatory studies in this setting. Additional studies in this setting that have the ability to account for potential confounders, including the use of lipid-modifying medications, would be of interest.

## Conclusions

Almost one-third of the Russia population has hypertriglyceridemia, defined as a TG level ≥1.7 mmol/L; this rate is not dissimilar to rates reported in Europe, the United States, and other countries. Although the prevalence of hypertriglyceridemia was higher in men than women, age-related increases in disease prevalence were observed for both sexes. The results of the PROMETHEUS study provide a robust and up-to-date estimate of the prevalence of hypertriglyceridemia, including severe forms of the disease, and help to define the size of the population that may require management of their disease. Correlations between TG and LDL-C, HDL-C, TC, and HbA1c (nested sample only) were observed.

## Abbreviations

ATP: adult treatment panel
CETP: cholesteryl ester transfer protein
CI: confidence interval
EAS: European Atherosclerosis Society
HbA1c: glycated hemoglobin
HDL-C: high-density lipoprotein cholesterol
LDL-C: low-density lipoprotein cholesterol
NCEP: National Cholesterol Education Program
NHANES: National Health and Nutrition Examination Survey
PCSK9: proprotein convertase subtilisin/kexin type 9
PO3FA: prescription omega 3 fatty acids
PPAR: peroxisome proliferator-activated receptor
PROMETHEUS: Prevalence of mixed dyslipidemia and severe hypertriglyceridemia in the Russian population
R^3^I: residual risk reduction initiative
RR: risk ratio
T2DM: type 2 diabetes mellitus
TC: total cholesterol
TG: triglyceride
VLDL: very low-density lipoprotein
